# All-Optical Single-Longitudinal-Mode Forward Brillouin Microwave Oscillator with an Unbalanced Fiber Mach–Zehnder Interferometer

**DOI:** 10.3390/mi16020209

**Published:** 2025-02-12

**Authors:** Xinyue Fang, Wenjun He, Wen Wang, Yi Liu, Yajun You, Qing Yan, Yafei Hou, Zepeng Wu, Lei Yu, Songquan Yan, Mingxing Li, Jian He, Xiujian Chou

**Affiliations:** 1 State Key Laboratory of Dynamic Measurement Technology, School of Instrument and Electronics, North University of China, Taiyuan 030051, China; 17860737007@163.com (X.F.); wangwen202308@163.com (W.W.); 15525490669@163.com (Q.Y.); houyafei_tl@163.com (Y.H.); wuzepeng2001@163.com (Z.W.); yulei13835520751@163.com (L.Y.); ysq17603593901@163.com (S.Y.); 19834538941@163.com (M.L.); drhejian@nuc.edu.cn (J.H.); chouxiujian@nuc.edu.cn (X.C.); 2 School of Aerospace Engineering, North University of China, Taiyuan 030051, China; 3 Thinvent Digital Technology Co., 681 Torch Avenue, High-Tech Industrial Development Zone, Nanchang 330096, China; 4 Shanxi Province Key Laboratory of Quantum Sensing and Precision Measurement, North University of China, Taiyuan 030051, China; yajunyou@nuc.edu.cn

**Keywords:** single-longitudinal-mode, forward Brillouin, Mach–Zehnder interferometer

## Abstract

An all-optical single-longitudinal-mode (SLM) forward Brillouin microwave oscillator (FB-MO) with an unbalanced Fiber Mach–Zehnder interferometer (UF-MZI) for microwave photonics (MWP) generation is proposed and experimentally investigated. UF-MZI consists of an optical coupler (OC), a polarization controller (PC), and two asymmetric length arms with 5 km and 500 m single-mode fibers (SMFs), which implements two unbalanced length feedback rings that are connected to one another. One long-length ring with a forward Brillouin gain cooperates with the other short-length ring to maintain a spectral Vernier effect and improve the effective free spectral range (FSR). By contrast with traditional optoelectronic oscillators (OEOs), this design does not require any photoelectric conversion devices and additional modulation, avoids external electromagnetic interference, and side-mode suppression and linewidth are favorable. Experimental results reveal that the 3-dB linewidth of the all-optical SLM FB-MO with UF-MZI is about 140 Hz. The acoustic-mode and side-mode suppression ratios are 26 dB and 31 dB. Within 60 min of the stability experiment, the power and frequency stability fluctuation were ±1 dB and ±100 Hz. Thanks to its long main ring cavity length, our all-optical SLM FB-MO with UF-MZI maintains good phase-noise performance. The measurement shows that a phase noise as low as −120 dBc/Hz at an offset frequency of 100 kHz is achieved. This SLM MWP generation technology holds great potential for applications in radar monitoring and wireless communication systems.

## 1. Introduction

At present, great progress in microwave photonics has been achieved in the fields of communication, radar, and sensing, including real-world applications [1,2,3]. Achieving high-speed transmission in wireless networks, as well as high-resolution detection in sensing systems and radar systems, requires microwave photon signals with narrow linewidth and low phase noise [4,5]. The common methods for generating narrow-linewidth microwave photon signals include the optical injection locking method [5,6], optical frequency comb method [7], optoelectronic oscillator [8,9], and mode-locked laser [10]. Although these methods can exhibit excellent phase noise, they also have certain limitations. OEO involves various optoelectronic devices such as optoelectronic modulators and amplifiers, which are susceptible to external electromagnetic interference, have poor stability, and result in limiting the application of OEO in certain aspects. The major limitation of using a mode-locked laser to generate photonic microwaves is that the microwave frequency has little tunability because it is determined by the longitudinal-mode spacing of the laser, which is fixed by the round-trip optical length of the laser cavity. The optical path structure of the optical frequency comb method is relatively complex and costly, while the optical injection locking method requires precise control of injection power and detuning, and the locking stability is greatly affected by the environment. Brillouin scattering has significant advantages in generating microwave photons and can produce microwave photon signals with stable frequency intervals [11]. In addition, the nonlinear effect of Brillouin scattering can easily narrow the linewidth of microwave photons [12]. However, the gain–bandwidth of the BSBS may be a limiting factor for improving the linewidth of MWPs, which is 30 MHz. Therefore, we focus on forward Brillouin scattering (FBS) in optical fibers and utilize its narrower gain–bandwidth compared to BBS to generate microwave photon signals with narrower linewidths [13]. As an internal optomechanical interaction in optical fibers, FBS introduces phase modulation during the process [14]. Therefore, we need a phase demodulation device to obtain FBS microwave photons. At present, the phase demodulation techniques for FBS to generate MWP include OEO with Sagnac loops and passive mode-locked fiber lasers [15,16,17,18,19,20]. A photoelectric RF oscillator has been reported that utilizes the FBS and the photoacoustic strong interaction in SMF to achieve a 318.7 MHz oscillating signal with 300 Hz narrow linewidth and approximately 40 dB acoustic mode suppression ratio [17]. An OEO based on FBS is demonstrated and proposed, which strictly limits the intense interaction in photonic crystal fiber (PCF) and achieves 1.237 GHz forward Brillouin oscillation with a 60 dB acoustic mode suppression ratio [18]. In addition, a wavelength tunable soliton fiber laser of 1.8 GHz is proposed by excitation of the base radial (R_01_) acoustic mode in the PCF core, which is the 389th harmonic of the cavity round trip frequency [19]. A soliton fiber laser that is passively mode-locked at the high harmonics of its intrinsic frequency inside the cavity is realized by using the strong-light mechanical interaction in a 60 cm short-length PCF using tight constraints of light and vibration [20]. However, the OEO and mode-locked lasers based on forward Brillouin scattering have also not overcome the disadvantages of electromagnetic interference and poor stability.

Previously, we proposed a forward-stimulated Brillouin microwave photon oscillator based on a fiber ring resonator, which utilizes the fiber ring resonator to control the FSR of the ring cavity, i.e., the Vernier effect, and generates narrow-linewidth microwave photons [21]. However, only the R_07_ acoustic mode was detected experimentally, and the tunability is poor. The fiber structure based on MZI can also effectively change the FSR of the ring cavity and provide a Vernier effect, which is widely used in narrow-linewidth lasers [22], high-resolution fiber optic sensing [23], and multi-wavelength lasers [24] and is used to implement mode selection [25,26,27]. A convex-shaped fiber ring-based SLM erbium-doped fiber laser is proposed, which is employed to serve as a high-quality mode filter to eliminate the dense longitudinal modes of the EDFL and attain the effective FSR of 12.58 GHz through the Vernier effect [25]. An EDF laser with a quad-ring is proposed and demonstrated, which can produce a mode-limited filter based on the Vernier effect and attain 2 kHz Lorentzian linewidth of the fiber laser [26]. A Mach–Zehnder experimental setup is reported and applied in the erbium-doped fiber (EDF) ring laser configuration that accomplishes the SLM oscillation with a narrow linewidth to the kHz target [27].

In this work, we demonstrate an all-optical SLM FB-MO with UF-MZI. During the process of oscillation, the oscillation frequency is locked by the forward Brillouin acoustic mode frequency and the FSR of the MZI ring cavity. Once the oscillation conditions are met, a high-quality microwave photon signal can be generated. The tunability of the frequency is also demonstrated by adjusting the polarization controller (PC). A tunable MWP ranging from 225 to 368 MHz with a frequency interval of about 50 MHz is obtained. Taking the R_07_ acoustic mode with the highest scattering efficiency as an example, test results show that the suppression ratios of the acoustic mode and side mode are 26 dB and 31 dB, respectively, and the linewidth at -3 dB is about 140 Hz. Additionally, power and frequency fluctuation within five hours are within ±1 dB and ±150 Hz, and the −120 dBc/Hz optimal phase-noise value is at a 100 kHz frequency offset.

## 2. Experiment and Principle

**Figure 1:**
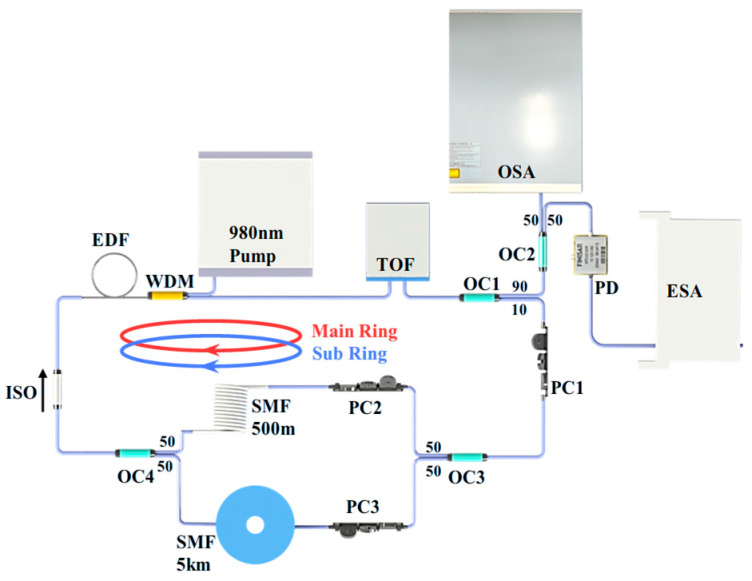
An all-optical SLM FB-MO with UF-MZI. WDM: wavelength division multiplexer; EDF: erbium-doped fiber; PC: polarization controller; OC: optical coupler; TOF: tunable filter; ISO: isolator; PD: photodetector; ESA: electrical spectrum analyzer; OSA: optical spectrum analyzer.

The structural diagram of an all-optical SLM FB-MO with UF-MZI is shown in Figure 1. Under the pumping of a 980 nm laser, the system utilizes a 20 m EDF as the gain medium to achieve laser gain, which serves as the pumping of FBS. A tunable optical filter (TOF) is employed to select the appropriate FBS pump wavelength. Additionally, an optical fiber isolator (ISO) ensures that the laser operates clockwise. FBS is excited by pump laser in a 5 km single-mode fiber, which is placed in a thermostat set at 25 °C with a resolution of ±0.2 °C to provide FBS in a controlled temperature environment and improve system stability. PC1 is used to adjust the polarization between Stokes waves and pump waves to ensure consistency and maximum FBS gain. In the measurement, the proposed MZI microwave oscillator configuration can result in two fiber rings, which mean the main and sub ring cavities (main ring cavity = 5 km and sub ring cavity = 500 m), as illustrated in Figure 1, cause the Vernier effect and achieve single longitudinal-mode output [24,25]; PC2 and PC3 are used to align the effective FSR of the main and sub ring cavities. Finally, the output laser is split into two beams through OC2, and one end of the OC2 is connected to an optical spectrum analyzer (OSA) to monitor the output laser’s spectrum with a resolution of 0.01 nm, while the other end is input into an electrical spectrum analyzer (ESA) with a resolution of 1 Hz after beating frequency by a photodetector (PD). The ESA is used to analyze the output laser’s frequency spectrum.

### 2.1. MWP Generation Principle of All-Optical SLM FB-MO with UF-MZI

**Figure 2:**
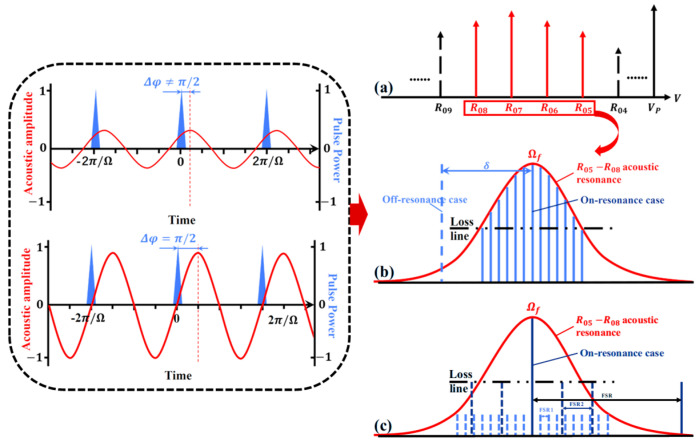
Production principle of all-optical SLM FB-MO with UF-MZI. The FBS acoustic mode gain spectrum of R_05_~R_08_ is depicted by the solid red line, with the driving pulse shown in blue. The solid blue line indicates resonance, while the dashed blue line represents non-resonance (dark blue for 500 m sub ring cavity and light blue for 5 km main ring cavity). (**a**) Different modes of forward Brillouin with the same spacing. (**b**) The oscillation mode of a ring cavity under forward Brillouin gain. (**c**) Single longitudinal mode output under cursor effect.

The principle of the all-optical SLM FB-MO with UF-MZI can be explained as follows. Forward Brillouin is excited by a driving pulse that excites the acoustic mode of forward Brillouin. The repetition rate of the driving pulse Ω is equal to an integer multiple of *FSR*_1_ (as shown in Figure 2b), and each optical pulse has a single pulse energy of *E_P_*. Forward Brillouin can exhibit R_05_~R_08_ modes, which correspond to different resonant frequencies $\Omega_{f}$ and are primarily determined by the cladding diameter of the SMF structure [28,29]. The offset between the repetition rate of the driving pulse and the resonant frequency of R_05_~R_08_ acoustic mode is expressed as $\delta =\Omega -\Omega_{f}$. We can express the acoustic wave generated by the driving pulse [30]:

(1) $${∆}_{\varepsilon_{r}}\left(z,t,r,\theta \right)=\gamma_{e}\frac{\rho }{\rho_{0}}=\frac{\gamma_{e}^{2}\left|Q\right|E_{p}\rho_{a}\left(r,\theta \right)e^{i\left(\Omega t-qz-∆\varphi \right)}}{4\pi n_{eff}cA_{eff}\rho_{0}\sqrt{4\delta^{2}+{\left({𝛤}_{B}\right)}^{2}}},$$

(2) $${∆}_{\varphi }=arccot\left(-2\delta /{𝛤}_{B}\right),\;0\leq ∆\varphi \leq \pi ,$$

where Δφ represents the relative phase shift between the sound wave and its driving pulse train, $\gamma_{e}$ is the electrostotion coefficient, $n_{eff}$ and $A_{eff}$ are the effective refractive index and mode area of the fundamental optical mode in SMF, c is the speed of light in a vacuum, and $\rho_{a}\left(r,\theta \right)$ is the dimensional acoustic profile of FBS acoustic mode. q is its propagation constant along the SMF axis, ${𝛤}_{B}$ is its Brillouin linewidth, and Q is the overlap integral between the electrostrictive stress field and the FBS acoustic mode [30]. As shown in Equation (1), the amplitude of the generated acoustic wave is inversely proportional to $\sqrt{4\delta^{2}+{\left({𝛤}_{B}\right)}^{2}}$, while the phase-matching condition requires that the propagation constant of the acoustic wave equals that of the driving pulses [31]. As shown in the dashed box in Figure 2, acoustic gain appears when the phase shift Δφ between the driving pulse train and the acoustic wave lies within the range (0, π) [32]. According to the Vernier effect, the effective FSR of the double-ring cavity structure is the least common multiple of the *R*_1_ and the *R*_2_, which is demonstrated in [33].

(3) $$FSR={n_{1}FSR}_{1}=n_{2}{FSR}_{2}$$

The *FSR*_1_ corresponds to the 5 km SMF main ring, and the *FSR*_2_ corresponds to the 500 m SMF sub ring, which is

(4) $${FSR}_{1}=\frac{c}{n_{e}L_{m}}(m=1,\;2)$$

where *c* represents the speed of light in a vacuum, and *n_e_* denotes the effective refractive index of the fiber circuit, which is 1.468. *FSR*_1_ and *FSR*_2_ correspond to the free spectral ranges of the two ring cavities formed by UF-MZI. In other words, the free spectral range is inversely proportional to the length of the fiber.

### 2.2. Linewidth of All-Optical SLM FB-MO with UF-MZI

In addition, the narrow-linewidth MWP output of the all-optical SLM FB-MO with UF-MZI is achieved by reducing the natural linewidth of the passive resonator. The intrinsic linewidth of the passive resonator is calculated as follows [34,35]:

(5) $$∆f=\frac{c}{nL}\frac{\left(1-K_{r}\right)}{\pi \sqrt{K_{r}}}$$

(6) $$K_{r}=(1-\gamma_{0})e^{-2\alpha_{0}L}$$

where *c* is the speed of light in vacuum, *L* is the length of the fiber ring cavity, *n* is the refractive index of SMF, $K_{r}=(1-\gamma_{0})e^{-2\alpha_{0}L}$ is the strength coupling coefficient, $\alpha_{0}$ is the amplitude attenuation coefficient of the fiber, and $\gamma_{0}$ is the insertion loss of the coupler. According to Equation (5), the longer gain fiber reduces the intrinsic linewidth ∆f of the passive resonator. After exciting FBS in the fiber, the linewidth of the Stokes light obtained further decreases. According to the linewidth theory of Brillouin scattering, we can derive the calculation formula for the linewidth of the all-optical microwave photons oscillator as follows [36]:

(7) $$∆v_{s}=\frac{∆f}{{\left(1+\gamma_{A}/\Gamma_{c}\right)}^{2}}=\frac{c(1-K_{r})}{nL{\pi \sqrt{K_{r}}(1+\frac{\pi {∆}_{B}}{-clnR/nL})}^{2}}$$

where $\gamma_{A}=\pi {∆}_{B}$ and $\Gamma_{c}=-clnR/nL$ represent the attenuation rate and the cavity loss rate of a sound wave, ${∆}_{B}$ is the FBS gain–bandwidth, and R is the optical amplitude feedback coefficient of the ring cavity.

## 3. Results and Discussion

### 3.1. SLM of R_07_-MWP

**Figure 3:**
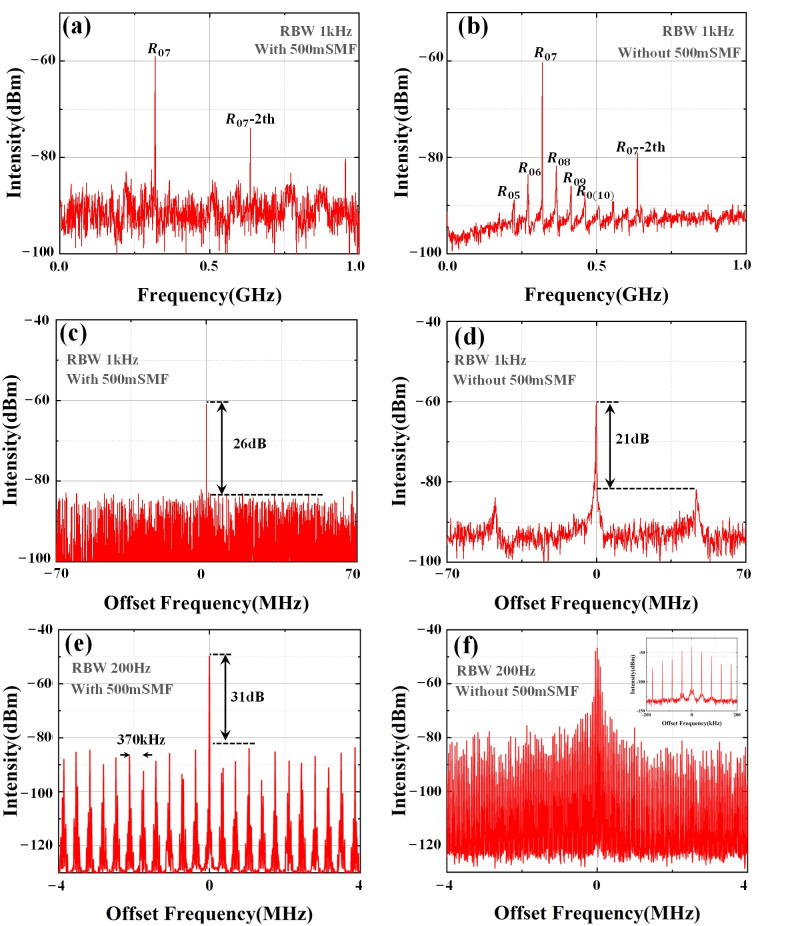
(**a**,**b**) show the heterodyne spectrum when the gain of R07 reaches its maximum with and without 500 m SMF (**c**,**d**) show the heterodyne spectrum with and without 500 m SMF at 140 MHz span. (**e**,**f**) are enlarged views at 8 MHz span.

First, we detected FBS acoustic modes of different frequencies in a 5 km main ring cavity by disconnecting the 500 m sub ring cavity. The results are shown in Figure 3b, with center frequencies corresponding to 220 MHz (R_05_), 270 MHz (R_06_), 318 MHz (R_07_), 365 MHz (R_08_), 414 MHz (R_09_), and 460 MHz (R_0(10)_). When the 500 m sub ring cavity is connected, as shown in Figure 3a, only R_07_-MWP and its higher-order modes can be seen in the spectrum. The maximum gain of R_07_ in Figure 3a,b is attained by fine tuning the PC. To demonstrate the effectiveness of the all-optical SLM FB-MO with UF-MZI, we compared the experimental results with and without the sub ring cavity using R07, which has the highest scattering efficiency. When connecting the 500 m sub ring cavity and reducing the spectrum range, we obtained a SLM microwave photon pulse oscillation at 318.08 MHz (R_07_), as shown in Figure 3c,e. Figure 3d,f display the spectrum of R_07_-MWP with only a 5 km SMF main ring cavity. By comparing (c) with (d), it can be observed that when both the 5 km main ring cavity and the 500 m sub ring cavity are present, the acoustic mode suppression ratio reaches 26 dB, which is approximately 5 dB higher than that without the 500 m sub ring cavity. The presence of main and sub ring cavities effectively suppresses longitudinal modes adjacent to R_07_-MWP, allowing for SLM operation as depicted in Figure 3e. Here, one can observe an *FSR*_2_ of about 370 kHz corresponding to the 500 m sub ring cavity, with an edge suppression ratio reaching 31 dB. Figure 3f depicts the R_07_-MWP of a main ring cavity, with the corresponding *FSR*_1_ of 43 kHz at 5 km illustrated. While the UF-MZI structure can achieve SLM output, it may have a certain impact on the linewidth. As per Equation (5), an increase in cavity loss will lead to a deterioration in the linewidth.

### 3.2. Linewidth of R_07_-MWP

**Figure 4:**
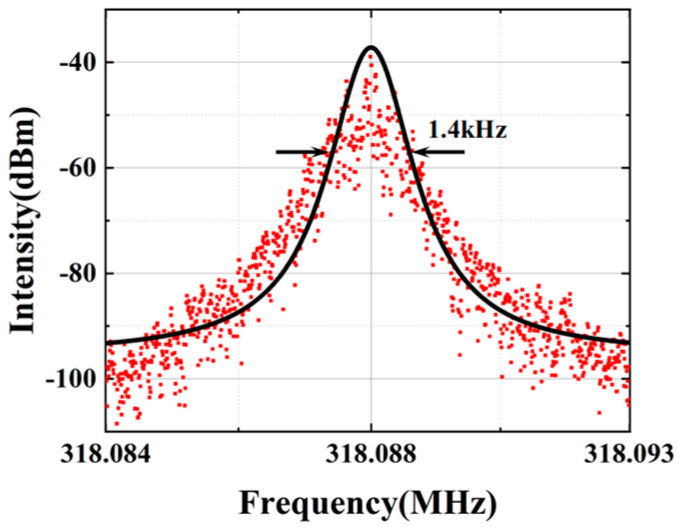
The linewidth of R_07_-MWP (with ESA measuring points indicated in red and the Lorentz fitting curve shown in black).

The linewidths of the R_07_-MWP are obtained from ESA measurements with 9 kHz spectral span, 5 Hz RBW, and VBW, as shown in Figure 4. Following Lorentz fitting, R_07_-MWP exhibits a linewidth of 1.4 kHz at the −20 dB power points, which is connected with the laser −3dB linewidth by √99 times. Therefore, the corresponding linewidth at the −3 dB point is about 140 Hz. Using Equation (7), we can roughly estimate the −3 dB linewidths of the MWPs. In the previous measurement, the FBS gain linewidth ${∆}_{B}$ is about 5.5 MHz. And the intra-cavity optical amplitude feedback coefficient R = 0.9. However, it should be noted that total loss of the ring cavity is 10 dB, so R′ = 0.5 × 0.5 × R = 0.674. In the 5 km ring cavity, the effective free spectrum is c/nl = 41 kHz, n = 1.4683, L = 5 km. The SMF attenuation of 0.18 dB/km implies a coupler insertion loss of $2\alpha_{0}L$ = 1.04, $K_{r}$ = 0.35. After calculating the value, the intrinsic linewidth of the passive resonator ∆f is 14.35 kHz [35], and we substitute it into Equation (5) to calculate the value of the real MWP linewidth $∆v_{s}$ ≈ 0.014 Hz. Due to the influence of system noise and unstable operation, the actual value deviates from the theoretical value.

### 3.3. Stability and Tunability Measurement

**Figure 5:**
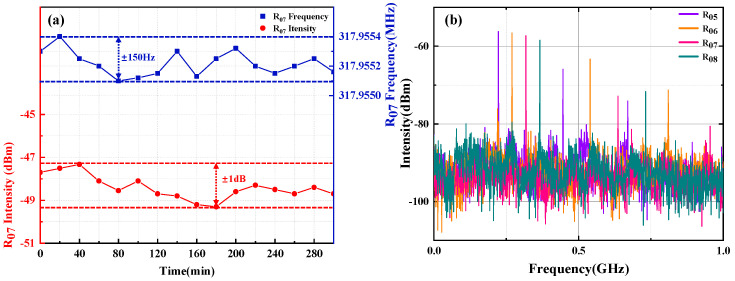
(**a**) Frequency stability and power stability of the R_07_-MWP and (**b**) frequency tunability of the oscillator.

Figure 5a illustrates the stability of the all-optical SLM FB-MO with UF-MZI. The RBW and VBW of the ESA is set as 100 Hz, and the SMF is placed in a 25 °C thermostat with a resolution of 0.2 °C. The power fluctuations and frequency fluctuations of R_07_-MWP can be seen in the range of ±1 dB and ±150 Hz for five hours. The frequency fluctuation range is less than 200 times that of *FSR*_1_, and there is no mode-hopping phenomenon within the expected range. In addition, by carefully adjusting the PC, we achieve the frequency tunability of the R_05_–R_08_-MWP, as shown in Figure 5b. R_05_–R_08_-MWP exhibits several relatively weak higher harmonics, satisfying multiples of the round-trip frequency in the cavity.

### 3.4. Phase Noise Measurement

**Figure 6:**
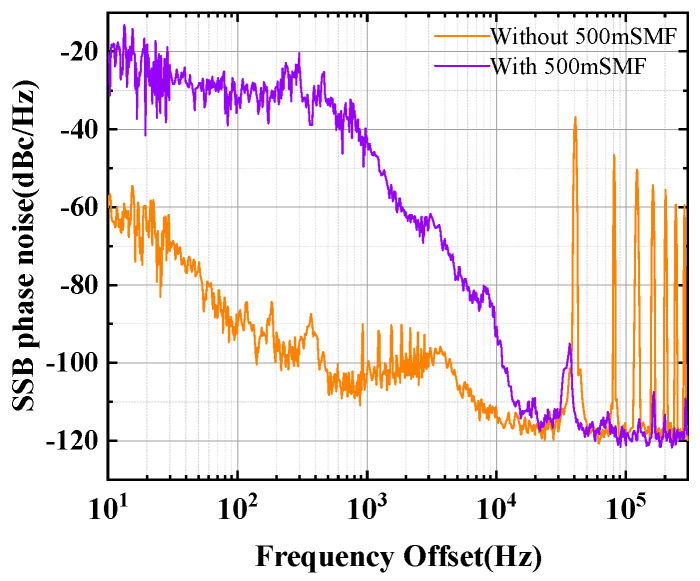
The SSB phase noise of R07-MWP.

The single sideband phase noise of the signal is measured by a spectrum analyzer (FSV3030 Rohde & Schwarz, Munich, Germany), and the results are shown in Figure 6. At a frequency offset of 100 kHz, all-optical SLM FB-MO with UF-MZI exhibits excellent phase-noise performance, achieving phase noise as low as −120 dBc/Hz. Due to the delay difference caused by different lengths of fiber arms in UF-MZI, interference occurs during the oscillation process of light inside the cavity, resulting in the deterioration of low-frequency phase noise [37]. At a frequency offset of 10 kHz, the phase noise with UF-MZI and without UF-MZI (only the 5 km main ring cavity exists) is −90.69 and −113.35 dBc/Hz. In addition, the interval of the resonance for the noise peaks presented in the trace without 500 m SMF is 43 kHz, corresponding to the free spectral range of the 5 km annular cavity. The presence of the resonance peaks can affect the measurement of microwave signals at resonance frequency, which is something we do not want to see. The resonance peak was well suppressed in the presence of 500 m SMF.

## 4. Conclusions

This article investigates the forward Brillouin single-longitudinal-mode microwave photon output under UF-MZI structure. Through different theoretical and experimental studies, it has been confirmed that forward Brillouin with smaller gain–bandwidth has significant advantages in generating narrow-linewidth microwave photons. In microwave oscillators based on forward Brillouin, we found a correlation between the intrinsic frequency of the resonant cavity and forward Brillouin acoustic mode frequency. If this condition is not followed, forward Brillouin cannot be fully excited. Therefore, the linewidth of the proposed all-optical SLM FB-MO with UF-MZI depends on the intrinsic linewidth of the passive resonant cavity and the Brillouin Stokes light. The traditional Vernier effect consists of two rings with slightly different lengths, used to achieve single-longitudinal-mode output. Unlike traditional Vernier effect schemes, the proposed scheme consists of two fiber arms with lengths that differ by km. The experimental results show that the proposed microwave oscillator has good stability, tunability, phase-noise performance, and extremely narrow linewidth. In addition, by utilizing special fibers such as photonic crystal fiber or highly nonlinear fibers, MWP at GHz and higher frequencies can be obtained, which also increases the cost of the device.

## Data Availability

Data are contained within the article.
